# Advancing neurofibromatosis research: a comparative review of animal models and their utility in dissecting the tumor microenvironment

**DOI:** 10.1186/s42826-026-00298-2

**Published:** 2026-09-20

**Authors:** Ai-Lun Li, Ya-Mei Chen

**Affiliations:** Department of Veterinary Medicine, College of Veterinary Medicine, National Pingtung University of Science and Technology, Pingtung, 912301 Taiwan

**Keywords:** 3Rs, Comparative medicine, Genetically engineered, Neurofibromatosis, Tumor microenvironment

## Abstract

Neurofibromatosis is a genetic disorder characterized by nervous system tumors arising primarily from *NF1* or *NF2* mutations. Given the complexity of the genetic background and heterogeneity of associated tumor phenotypes in neurofibromatosis, mechanistic investigations rely heavily on experimental animal models. Human tissue-based studies alone cannot fully elucidate causal relationships, temporal dynamics, modifier gene effects, or cell type-specific contributions to tumor development, progression, and microenvironmental regulation. Consequently, various animal models have been developed, including genetically engineered mouse models, large-animal systems, alternative vertebrate models, and transplantation-based approaches. These experimental platforms enable the controlled manipulation of gene dosage, lineage-specific gene deletion, immune modulation, targeted disruption of stromal and extracellular matrix components, and evaluation of therapeutic response and drug resistance mechanisms. Animal models have helped demonstrate the cell-autonomous and non-cell-autonomous mechanisms underlying neurofibromatosis tumorigenesis. Furthermore, sustained tumor maintenance and progression require coordinated interactions between neoplastic cells and their surrounding microenvironment. Notably, these experimental systems offer a temporal resolution not achievable in clinical specimens, demonstrating that microenvironmental alterations may precede overt tumor formation. Such findings depict the tumor microenvironment (TME) as an active contributor to disease rather than merely as a consequence of tumor growth. In this review, we evaluate the experimental scope, methodological strengths, and inherent limitations of current animal models used in neurofibromatosis research, with emphasis on their utility in elucidating interactions within the TME and non-neoplastic disease manifestations. This work characterizes model-specific advantages and constraints to emphasize the continued importance of animal models as essential tools for hypothesis-driven investigation and translational development in elucidating the various attributes of neurofibromatosis.

## Background

Neurofibromatosis is a genetic disorder characterized by tumors developing predominantly within the nervous system. It is mainly divided into neurofibromatosis type 1 (NF1) and neurofibromatosis type 2 (NF2) [1, 2]. NF1 is caused by mutations in *NF1*, which is located on chromosome 17q11.2 and encodes neurofibromin, a negative regulator of Ras signaling [1, 2]. Loss of neurofibromin function contributes to dysregulated cell proliferation and the development of peripheral nerve sheath tumors, including cutaneous and plexiform neurofibromas [3]. NF1 affects approximately 1 in 2,500–3,000 individuals and is frequently associated with additional manifestations involving the skin (café-au-lait macules, axillary/inguinal freckling, and cutaneous neurofibromas), skeletal system (scoliosis, tibial dysplasia, and sphenoid wing dysplasia), or nervous system (optic pathway gliomas, other central and peripheral nervous system tumors, and neurocognitive impairment) [4, 5]. The clinical diagnostic criteria for NF1 were first established by the National Institutes of Health in 1988 and revised in 2021 to improve diagnostic accuracy [6–8]. NF1 is diagnosed when an individual meets two or more criteria, including café-au-lait macules, axillary or inguinal freckling, neurofibromas or plexiform neurofibroma, optic pathway glioma, Lisch nodules or choroidal abnormalities, distinctive osseous lesions, or a heterozygous pathogenic NF1 variant with an approximately 50% variant allele fraction in apparently normal tissue. Other concurrent features, such as learning disabilities, attention-deficit/hyperactivity disorder, and behavioral problems, are common. Vascular abnormalities (e.g., hypertension, moyamoya vasculopathy) and increased malignancy risk also contribute to morbidity [4, 5].

In contrast, NF2 is a rarer autosomal dominant disorder, with a prevalence of approximately 1 in 25,000–33,000 individuals [9–11]. It is characterized by germline mutations in *NF2*, which is located on chromosome 22q12 and encodes the tumor suppressor protein merlin (also known as schwannomin) [9]. Loss of merlin function facilitates the development of multiple benign tumors, most notably bilateral vestibular schwannomas, but also meningiomas, ependymomas, and other nervous system lesions [9, 12, 13]. NF2 is clinically characterized by the development of multiple central nervous system tumors, most notably bilateral vestibular schwannomas, followed by meningiomas and ependymomas, which contribute to significant morbidity [12]. NF2 phenotypic expression is highly variable. Approximately half of cases arise from de novo mutations, with mosaicism commonly manifesting among sporadic presentations [10, 14]. Although NF1 and NF2 are genetically and clinically distinct, overlapping features occasionally occur, demonstrating the complexity of tumorigenesis within the nervous system [9, 15].

Given the well-defined genetic basis of neurofibromatosis, along with its diverse tumor phenotypes and complex interactions between neoplastic cells and their microenvironment, this disorder is particularly suitable for investigation using experimental animal models. Models established using genetically engineered mice are central to elucidating disease mechanisms that cannot be fully examined through human tissue studies alone, especially when investigating tumor initiation, progression, and tumor microenvironment (TME) interactions.

## Main text

### Tumor cell-intrinsic pathogenesis

Although schwannomas, neurofibromas, and malignant peripheral nerve sheath tumors (MPNSTs) all develop from the Schwann cell lineage, mounting evidence demonstrates that these neoplasms are driven by distinct pathogenic mechanisms. Comparative analyses between human tumors and genetically engineered mouse models (GEMMs) reveal that NF1- and NF2-associated tumors differ not only in their initiating genetic events, but also in their downstream signaling dependencies, cooperating tumor suppressor alterations, and interactions with non-neoplastic cell populations [16].

Neurofibromatosis arises primarily from *NF1* or *NF2* mutations, with *NF1* mutations accounting for most cases studied in experimental models [17]. *NF1* encodes for neurofibromin, a GTPase-activating protein (GAP) that negatively regulates Ras signaling and constrains downstream pathways, including the Ras/MAPK and AKT/mTOR cascades [18]. Loss of neurofibromin function causes sustained Ras activation, promoting aberrant cell proliferation and survival, thereby initiating neurofibroma development [19]. Biallelic loss of *NF1* in Schwann cell lineage constitutively activates the Ras pathway, increasing cell proliferation and dedifferentiation, which underlie the formation of both cutaneous and plexiform neurofibromas [18]. Plexiform neurofibromas develop from Schwann cells after biallelic NF1 loss, often during embryonic development when Schwann cell precursors are abundant and more susceptible to transformation [20]. However, with biallelic *NF1* loss, cutaneous neurofibromas arise from non-myelinating Schwann cells after postnatal biallelic NF1 loss, and these tumors mayproliferate in response to local trauma [3]. Both tumor types exhibit delayed senescence, loss of density-dependent growth inhibition, and increased extracellular matrix (ECM) production, sustaining their neoplastic potential [3, 21]. Progression to MPNSTs can occur through additional genetic alterations, including CDKN2A loss, RB1 loss, TP53/p53 inactivation, PTEN loss, and epigenetic dysregulation [22–24]. These alterations further promote cell-cycle deregulation, genomic instability, Ras/MAPK and PI3K/AKT/mTOR pathway activation, and malignant transformation [22–24].

Beyond its role in Ras regulation, neurofibromin modulates cytoskeletal organization through the Rho-ROCK-LIMK2-cofilin pathway, which influences actin filament dynamics, cell motility, and adhesion [25–27]. Loss of neurofibromin increases cytoskeletal remodeling and altered cell migration through RhoA/ROCKdependent signaling pathways, resulting in excessive actin polymerization, altered cellular morphology, and enhanced cell motility [25]. In addition to the canonical regulation of classic Ras proteins, neurofibromin also functions as a GTPase-activating protein for the R-Ras subfamily [28]. Recent studies have demonstrated that R-Ras and R-Ras2 are activated in neurofibromin-deficient MPNST cells and contribute to tumor progression by regulating signaling networks involved in cytoskeletal remodeling, vesicular trafficking, migration, and invasion [28]. Studies on siRNA-mediated knockdown of neurofibromin in cell models have demonstrated that neurofibromin deficiency induces morphological changes characterized by increased actin stress fibers and upregulated phosphorylation of cofilin, a key regulator of actin filament dynamics. This process is mediated by the activation of GTPase activation and downstream ROCK signaling, independent of the canonical Ras-Raf-MEK-ERK pathway [25]. Furthermore, inhibition of R-Ras signaling reduces migration and invasion while decreasing ROCK1 and MYPT1 phosphorylation, supporting a mechanistic link between neurofibromin loss, R-Ras activation, and ROCK-dependent cytoskeletal regulation [28]. These alterations are directly linked to increased cell migration, invasion, and tumor progression in NF1-associated neoplasms [28]. In MPNST cells, R-Ras signaling appears to regulate migratory behavior through RhoA–ROCK1-mediated pathways that are distinct from those controlled by classic Ras proteins [28]. Furthermore, neurofibromin deficiency promotes epithelial–mesenchymal transition (EMT) signaling, which increases the migratory and invasive potential of tumor cells [29]. Recent evidence also suggests that lysophosphatidic acid (LPA) signaling may serve as an upstream activator of this migratory program [30]. MPNST cells predominantly express LPAR1 and aberrantly express LPAR3, in contrast to normal Schwann cells, which mainly express LPAR1 and LPAR6 [30]. LPAR1 mediates LPA-induced migration through activation of R-Ras/R-Ras2 and downstream ROCK signaling, whereas LPAR3 primarily promotes tumor cell proliferation. Genetic or pharmacologic inhibition of LPAR1, R-Ras, or ROCK effectively suppresses MPNST cell migration, highlighting the importance of the LPAR1–R-Ras–ROCK axis in MPNST pathogenesis [30]. Post-translational modifications of neurofibromin, such as O-glycosylation, further modulate the functional interactions and regulatory capacity of neurofibromin [31]. Specifically, O-GlcNAcylation (a form of O-glycosylation) and phosphorylation can occur on the same or adjacent serine/threonine residues, referred to as “yin-yang” sites, creating a regulatory interplay that fine-tunes the Ras-GAP activity and downstream signaling of neurofibromin. This crosstalk can either promote or inhibit the ability of neurofibromin to suppress Ras signaling, depending on the balance of modifications, thus directly affecting cell proliferation and migration relevant to tumorigenesis in NF1 [31]. Altered O-glycosylation may disrupt interactions of neurofibromin with key signaling partners, including those in the Ras/MAPK and Akt/mTOR pathways, and can influence its subcellular compartmentalization, thereby further modulating its tumor suppressor function [32]. Dysregulation of these post-translational modifications has been implicated in the loss of neurofibromin function, thereby increasing Ras activityand potentially cooperating with R-Ras/ROCK-mediated signaling pathways to enhance cell motility and tumor progression [31, 32]. However, the mechanistic evidence supporting the role of R-Ras–ROCK signaling in MPNST migration and invasion is currently more direct than that available for post-translational modifications of neurofibromin.

In MPNSTs, which are an aggressive complication of NF1, hyperactivated Ras signaling induces concurrent activation of the AKT/mTOR and MAPK pathways [22–24]. Experimental studies on *Nf1*-mutant mouse models, either alone or in combination with other genetic alterations, have revealed the contributions of these signaling axes to malignant transformation and tumor progression [33]. Activation of these pathways contributes to increased tumor growth and reduced therapeutic responsiveness, providing mechanistic insights that are difficult to obtain from human samples alone [22–24, 33]. Genetic modeling approaches have further demonstrated that loss of *Nf1* heterozygosity is critical to neurofibroma development and malignant progression [33]. Chimeric mouse and compound mutant models incorporating alterations in other tumor-associated genes, such as *Trp53*, have been used to elucidate the synergistic effects of genetic modifiers in driving MPNST development [33]. The NPcis mouse (Nf1^+/−^;Trp53^+/−^ cis) carries null Nf1 and Trp53 alleles linked in cis on chromosome 11; tumors frequently undergo concurrent loss of the wild-type alleles, and the mice spontaneously develop MPNSTs and astrocytomas [33].

Another important peripheral nerve sheath tumor model is the P_0_-GGFβ3;*Trp53*^+/−^ mouse, which combines Schwann cell-directed overexpression of glial growth factor β3 (GGFβ3), a type II NRG1 isoform driven by the myelin protein zero (P0/Mpz) regulatory sequence, with Trp53 haploinsufficiency while retaining wild-type *Nf1* [34]. This model develops high-penetrance de novo MPNSTs on an inbred C57BL/6J background, demonstrating cooperation between NRG1/ErbB signaling and p53 insufficiency rather than biallelic Nf1 loss [34]. It is therefore useful for studying alternative molecular routes to MPNST development, although its intact Nf1 and absence of a consistent benign neurofibroma precursor limit its genetic fidelity to NF1.

Beyond tumorigenesis, *Nf1* haploinsufficiency affects neural circuit function. *Nf1*^⁺/⁻^ mice used in behavioral models exhibit abnormalities in learning, attention, and social behavior, which have been linked to alterations in GABAergic neurotransmission induced by Ras hyperactivation, with additional involvement of dopaminergic regulation [35, 36]. In addition to Ras hyperactivation, neurofibromin also acts as a positive regulator of cAMP signaling in the nervous system. Loss of NF1 can therefore reduce cAMP-mediated signaling in the brain, contributing to neurodevelopmental and behavioral phenotypes observed in NF1 models [37, 38].

The pathogenesis of neurofibromatosis type 2 is driven by loss-of-function mutations in *NF2*, which is located on chromosome 22q12 and encodes the tumor suppressor protein merlin (also known as schwannomin). Merlin is structurally related to the ezrin-radixin-moesin family and is widely expressed in Schwann cells, meningeal cells, and other nervous system tissues [39, 40]. Normally, it regulates cell proliferation, maintains contact-dependent growth inhibition, and modulates key signaling pathways, such as PI3K/AKT, Raf/MEK/ERK, and mTOR [39, 40]. Tumor formation in NF2 follows the Knudson “two-hit” hypothesis: affected individuals inherit one mutated *NF2* allele, whereas a somatic mutation in the second allele in Schwann cells or other relevant cell types induces loss of merlin function and subsequent tumorigenesis [41]. Loss of merlin disrupts cytoskeletal organization, cell–cell adhesion, and growth suppression, causing the uncontrolled proliferation of Schwann cells and meningeal cells, which manifest clinically as bilateral vestibular schwannomas, meningiomas, and other nervous system tumors [14, 42]. Merlin also acts upstream of the Hippo tumor suppressor pathway to regulate cell growth and apoptosis. In contrast, its inactivation induces the dysregulation of these pathways, promoting benign tumor development and associated morbidity [40, 42]. The clinical variability in NF2 is influenced by the type and location of *NF2* mutations, with truncating mutations generally causing greater disease severity.

### Animal models applied in neurofibromatosis research: Scope, strengths, and limitations

Animal models have contributed significantly to neurofibromatosis research by enabling experimental analyses that are not feasible in human studies. Given the genetic complexity of the disease and the diversity of associated tumor phenotypes, no single model can recapitulate all aspects of neurofibromatosis. Thus far, a broad range of experimental systems has been developed, each with distinct strengths and limitations (Table 1). These include GEMMs, large-animal models, alternative vertebrate models, and transplantation-based approaches. This section provides an overview of these animal models, focusing on their experimental scope and suitability for investigating specific biological questions, rather than on detailed pathogenic mechanisms. For clarity, the 3Rs principles refer to Replacement, Reduction, and Refinement. In this review context, the 3Rs are considered in relation to model selection, experimental efficiency, avoidance of unnecessary animal use, and refinement of disease-relevant endpoints.

**Table 1:** Comparative animal models of neurofibromatosis and NF-related disease across species

Species / Model	Model type	Genetic alteration	Key cell types emphasized	Major phenotypes reproduced	Key use-case / limitation	Key references
Mouse (NPcis)	Genetically engineered (compound heterozygote)	Nf1^+/−^;Trp53^+/−^ null alleles linked in cis on chromosome 11	Neural crest/Schwann lineage and glial cells	Spontaneous MPNSTs and astrocytomas; concurrent Nf1/Trp53 LOH	Malignant transformation and modifier studies / Benign neurofibroma precursor is inconsistent	[33,51]
Mouse (P0-GGFβ3;Trp53^+/−^)	Transgenic/compound heterozygote	P0(Mpz)-driven GGFβ3(NRG1) overexpression; Trp53^+/−^; Nf1^+/+^	Myelinating Schwann cells/peripheral glia	High-penetrance de novo MPNSTs; no consistent benign neurofibroma precursor	NRG1/ErbB–p53 cooperation / Intact Nf1 limits NF1 genetic fidelity	[34]
Mouse (NF1 Cre-Lox GEMM)	Genetic engineered (conditional KO)	*Nf1* flox with tissue-specific Cre-Lox	Schwann cells, astrocytes, bone marrow, chondrocytes	Plexiform/cutaneous neurofibroma, OPG, MPNSTs, skeletal dysplasia	Best for cell-of-origin/mechanism Limited human-scale translation	[17,20,45–47,49,52–56,91]
Mouse (germline or knock-in NF1)	Genetic engineered	Germline *Nf1*^+/−^ or patient-relevant variants	System-level	Systemic and neurobehavioral abnormalities Low-frequency tumor formation	Best for gene dosage and modifier studies Limited faithful tumor recapitulation	[17,35,36,43,44,57]
Mouse (NF2 GEMM)	Genetic engineered (conditional KO)	*Nf2* flox with Schwann cell–specific Cre-Lox	Schwann lineage	Schwannomas, hearing/balance deficits	Functional NF2 modeling Mouse-specific biology	[16,43,44,66–68]
Minipig (NF1)	Gene-edited large animal	Germline heterozygous *Nf1* pathogenic variant Spontaneous LOH reported	Schwann lineage + melanocytes	CALMs, cutaneous neurofibromas, optic pathway lesions, skeletal/behavioral phenotypes	Human-like imaging/PK/PD High cost and limited availability	[61–64]
Dog	Spontaneous PNSTs	Sporadic or inherited predisposition (often not fully genotyped)	Peripheral nerve sheath + intact immune/TME	PNSTs, MPNSTs Progression may be observable	High clinical realism Molecular heterogeneity and limited standardization	[61]
Cattle	Spontaneous/herd-associated	Heritable susceptibility suggested (Holstein neurofibromatosis)	Peripheral nerve sheath	Multiple neurofibromas/PNST-like lesions	Useful for inherited susceptibility concept Scattered data and limited mechanistic understanding	[61]
Zebrafish	Genetic engineered or case-based	varies	varies	pathway-level phenotypes	Screening/rapid genetics Lower clinical face validity	[37,65]

Abbreviations: CALMs, café-au-lait macules; GEMM, genetically engineered mouse model; KO, knockout; LOH, loss of heterozygosity; MPNSTs, malignant peripheral nerve sheath tumors; NF1, neurofibromatosis type 1; NF2, neurofibromatosis type 2; OPG, optic pathway glioma; PD, pharmacodynamics; PK, pharmacokinetics; PNSTs, peripheral nerve sheath tumors; GGFβ3, glial growth factor beta 3; NRG1, neuregulin 1; P0/Mpz, myelin protein zero

Mouse models are the most widely used experimental systems in neurofibromatosis research, particularly for NF1 [43, 44]. GEMMs, which are most commonly based on Cre-Lox-mediated conditional deletion of *Nf1*, provide precise spatial and temporal control over gene loss and are therefore suitable for investigating cell lineage-specific requirements, developmental timing, and stepwise tumor progression [45–49]. GEMMs have been used to reproduce major NF1-associated tumor types, including plexiform and cutaneous neurofibromas, optic pathway gliomas, and MPNSTs [17, 20, 45–49]. Depending on the genetic strategy employed, GEMMs can help elucidate questions related to cell of origin, the requirement for additional genetic alterations, and the consequences of targeted gene loss within defined cellular compartments [17, 20, 45–49]. From an experimental design perspective, conditional mouse models also refine the 3Rs framework by minimizing off-target effects and unnecessary animal burden. It is important to note, however, that NF1 alterations in humans and animal models do not always represent complete gene knockout [50]. Some genomic mutations result in reduced NF1 expression or hypomorphic function, whereas others involve conditional or lineage-specific biallelic deletion. Recent reassessment of NPcis mice also identified rare neurofibroma-like lesions, highlighting the influence of genetic background and systematic histopathologic sampling on model phenotypes [51]. Complete Nf1 knockout in mice is embryonic lethal, typically between embryonic days 11.5 and 13.5, underscoring the need to select models according to the specific genetic state and disease stage being investigated [33].

Multiple categories of NF1-related mouse models have been developed, including germline or conditional *Nf1* mutant mice, chimeric models, and compound mutant models incorporating additional cancer-associated genetic alterations [17, 33, 45, 46, 49, 51]. A key strength of GEMMs is that they facilitate the precise manipulation of *Nf1* loss at the spatial and temporal scales relevant to defined cell lineages, thereby enabling the systematic investigation of cell of origin hypotheses and stage-specific requirements during tumor development. These models allow the controlled assessment of the effects of *Nf1* loss in Schwann cell lineages on neurofibroma initiation, as well as the influence of additional genetic events, such as *Ink4a/Arf* deletion, on progression toward atypical neurofibroma and MPNSTs [17, 47, 49]. Furthermore, *Nf1* mutant mouse models reproduce several clinically relevant disease features, such as the development of optic pathway gliomas, providing tractable systems for studying tumor initiation, progression, and associated neurological dysfunctions under defined genetic conditions [52–54]. Collectively, these attributes make GEMMs particularly suitable for the mechanistic dissection of tumor development pathways and for the hypothesis-driven evaluation of candidate therapeutic interventions.

In addition to tumor modeling, conditional and germline *Nf1* mouse models have been widely used to investigate non-neoplastic manifestations of NF1, particularly skeletal and neurodevelopmental abnormalities. Osteoblast- or osteoclast-specific *Nf1* conditional knockout mice successfully recapitulate characteristic skeletal pathologies, including tibial bowing, pseudarthrosis, and decreased bone density [55, 56]. Furthermore, these mouse models have demonstrated that *Nf1* loss disrupts normal bone remodeling by impairing osteoblast differentiation, altering pyrophosphate metabolism, and activating secondary osteoclasts [55, 56]. Beyond skeletal phenotypes, germline or heterozygous *Nf1* mutant mice have been used to study the neurological and behavioral manifestations of NF1, including learning, attention, and social behavior deficits [35, 36, 57]. Despite not fully capturing the clinical heterogeneity observed in human patients, these models provide experimentally tractable systems for integrating molecular, cellular, and behavioral analyses in an immune-competent setting. From a 3Rs standpoint, conditional mouse models refine experimental design by enabling lineage- and time-specific gene manipulation, thereby reducing phenotypic variability and the number of animals required to test defined mechanistic hypotheses.

Transplantation-based models, including patient-derived xenografts (PDX) and orthotopic xenograft systems, involve engrafting human neurofibroma-derived Schwann cells into immunodeficient mice [58, 59], thereby enabling the in vivo assessment of human tumor cell behavior under controlled experimental conditions. However, the requirement for immunocompromised hosts limits the utility of such models for investigating immune-mediated processes and TME interactions in a fully physiological setting [46, 58]. Orthotopic and transplantation-based models help refine studies by allowing longitudinal functional and therapeutic assessment within anatomically relevant contexts, thereby reducing reliance on large cohorts and terminal experimental endpoints.

Despite their versatility, mouse models have inherent limitations stemming from species-specific biology and genetic diversity [60]. Furthermore, engineered loss of heterozygosity does not always reflect the spontaneous mutational processes occurring in human neurofibromatosis [61]. These limitations have prompted the development of complementary large-animal models. Porcine (minipig) models have emerged as valuable large-animal systems for neurofibromatosis research, offering greater anatomical and physiological similarities to humans than rodent models. Genetically engineered NF1 minipigs, such as those with *NF1*^*+/ex42del*^, recapitulate multiple hallmark features of human NF1, including café-au-lait macules, neurofibromas, axillary freckling, and skeletal abnormalities. They are also amenable to longitudinal assessments and advanced imaging approaches [62–64]. These features make minipig models particularly suitable for translational research and preclinical therapeutic evaluation [61]. Despite their high cost and infrastructure demands, the superior anatomical and physiological fidelity of porcine models enhances predictive validity and supports translational reduction by limiting the need for repeated downstream animal studies. Reports of spontaneous NF-like tumors in other large animals, such as dogs and cattle, remain limited. Furthermore, these species are currently used primarily as comparative models rather than as standardized experimental systems [61].

Alternative vertebrate models, such as zebrafish carrying mutations in the *NF1* orthologs *nf1a* and *nf1b*, provide complementary platforms for studying conserved aspects of NF1 biology [65]. Zebrafish models are particularly advantageous for genetic manipulation, developmental studies, and rapid in vivo screening [65]. Nevertheless, their anatomical and immunological differences from mammals restrict their applicability for modeling complex TME and clinical disease phenotypes [65]. Alternative vertebrate models facilitate reduction by enabling rapid genetic and pharmacological screening, thereby prioritizing candidate pathways before validation in mammalian systems.

In contrast to NF1, fewer GEMMs faithfully recapitulate the full anatomical and functional spectrum of NF2-associated tumors. Early *Nf2*^*+/−*^ mouse models exhibit clear species-specific limitations, as these animals predominantly developed osteosarcomas rather than the schwannomas characteristic of human NF2 [43, 44, 66]. Subsequent studies have demonstrated that conditional, biallelic inactivation of *Nf2* within the Schwann cell lineage is necessary to reproduce key NF2 manifestations, confirming that inadequate second allele loss underlies the failure of hemizygous models [66, 67]. Early Schwann cell-specific Cre systems provided initial proof of principle but showed variable penetrance and limited functional characterization [16]. A major development was achieved with *Postn-Cre*; *Nf2*^*flox/flox*^ mice, which develop schwannomas affecting cranial, spinal, and peripheral nerves. This model exhibits complete penetrance and histopathological features indistinguishable from those of human disease, accompanied by measurable hearing and balance deficits [67]. The resulting tumors are histologically indistinguishable from human schwannomas [67]. These models have since enabled integrated anatomical, functional, and preclinical therapeutic studies of NF2 [68].

Studies targeting p21-activated kinases (PAKs) in merlin-deficient NF2 mouse models have shown that genetic ablation of Pak1, but not Pak2, reduces tumor burden and extends survival, underscoring the capacity of these models to reveal isoform-specific therapeutic dependencies that are difficult to clarify in human studies alone [68]. Along with genetically engineered approaches, anatomically targeted transplantation-based models have been developed to address functional limitations in NF2 research [69]. Orthotopic implantation of schwannoma cells into the cerebellopontine angle (CPA) region facilitates investigation of vestibular schwannoma growth in a clinically relevant anatomical context and supports longitudinal assessment of hearing loss, balance impairment, angiogenesis, and therapeutic responses [69]. Subsequent refinement of this CPA-based model has further expanded its functional scope by integrating quantitative assessments of ataxia and motor coordination [70]. Using a standardized panel of gait, balance, and coordination tests combined with auditory measurements, recent studies have demonstrated that tumor progression may be accompanied by progressive vestibular and motor dysfunction [70]. Furthermore, therapeutic interventions can differentially ameliorate these neurological deficits alongside tumor management [70]. These findings demonstrate the utility of orthotopic NF2 models in the comprehensive evaluation of tumor-induced neurological impairment and therapeutic efficacy beyond tumor size alone [69, 70]. Although such models do not recapitulate tumor initiation, they provide complementary platforms for studying neurological consequences and treatment effects that are otherwise challenging to capture in conventional GEMMs [69, 70].

**Fig. 1:**
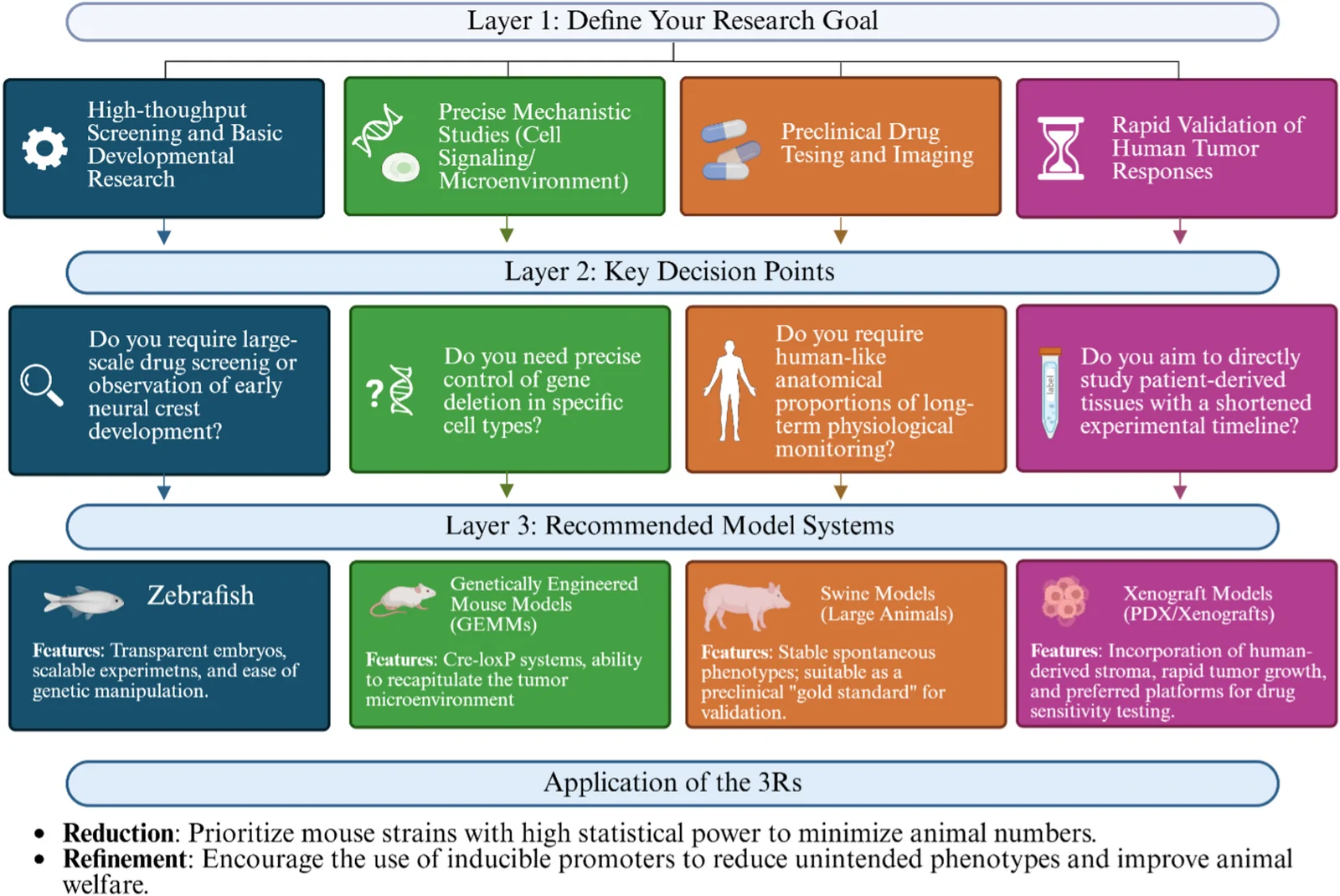
Decision pathway for selecting experimental models in NF1 research. This schematic presents a goal-driven framework that matches specific research objectives to appropriate NF1 model systems. Based on key experimental requirements, zebrafish, genetically engineered mouse models, large-animal (swine) models, or patient-derived xenografts are currently recommended to balance mechanistic resolution, translational relevance, and adherence to pathological rigor and 3Rs principles

Together, these experimental systems constitute a complementary toolkit for neurofibromatosis studies. The selection of the appropriate animal model should be guided by the specific biological question being addressed, along with careful consideration of the experimental scope and inherent limitations of each model (Fig. 1).

### TME in neurofibromatosis animal models

The TME is a crucial focus of studies on neurofibromatosis-associated tumor development and progression; however, the causal relationships among genetic alterations, stromal components, and immune populations cannot be fully expounded using human tissue alone. Limitations in sample accessibility, temporal resolution, and experimental manipulation restrict the scope of clinical studies in dissecting dynamic TME interactions. In this context, experimental animal models provide indispensable platforms for the functional interrogation of TME components and for establishing causal links between microenvironmental factors and tumor behavior.

GEMMs facilitate systematic testing of non-cell-autonomous contributions to NF1-associated tumorigenesis within in vivo experimental systems [17, 45, 71, 72]. Mouse models also enable the systematic evaluation of stromal contributions to tumor architecture and progression [73]. Genetic and pharmacologic manipulation of fibroblasts and ECM components facilitates assessment of how matrix remodeling influences tumor organization, invasion, and vascularization [73]. These experimental systems permit controlled perturbation of collagen deposition, basement membrane composition, and matrix-associated signaling pathways, thereby revealing functional dependencies that cannot be readily uncovered in human specimens [73].

Immune cell populations are another major component of the TME that can be selectively interrogated using animal models [74–76]. Bone marrow transplantation and genetic or pharmacologic manipulation enable direct testing of macrophage- and mast cell-associated contributions to tumor-associated processes and longitudinal analysis of immune–tumor interactions [74–76]. Furthermore, animal models support functional analysis of cytokine- and growth factor-associated signaling networks within the TME [75, 77, 78]. In vivo perturbation of inflammatory and stromal signaling pathways enables evaluation of their roles in immune-cell recruitment and tissue remodeling [75, 77, 78].

Despite these advances, species-specific differences in immune composition, stromal biology, and tumor kinetics necessitate cautious interpretation when extrapolating findings to human disease. Nevertheless, animal models remain indispensable for elucidating the causal relationships underlying TME-dependent tumor behavior.

### Relationship between neurofibromatosis and TME in animal models

Insights from diverse animal models collectively indicate that neurofibromatosis-associated tumors are best viewed as microenvironment-dependent disease systems, rather than purely tumor-cell-autonomous neoplasms [71, 74, 77, 79]. Across experimental platforms, tumor initiation, progression, and associated clinical manifestations consistently develop from dynamic, reciprocal interactions between genetically altered cells and their surrounding host tissues [71, 74, 77, 79]. These findings have reshaped the conceptual framework of neurofibromatosis, highlighting the TME as an essential determinant of disease behavior rather than a secondary byproduct of tumor growth.

**Fig. 2:**
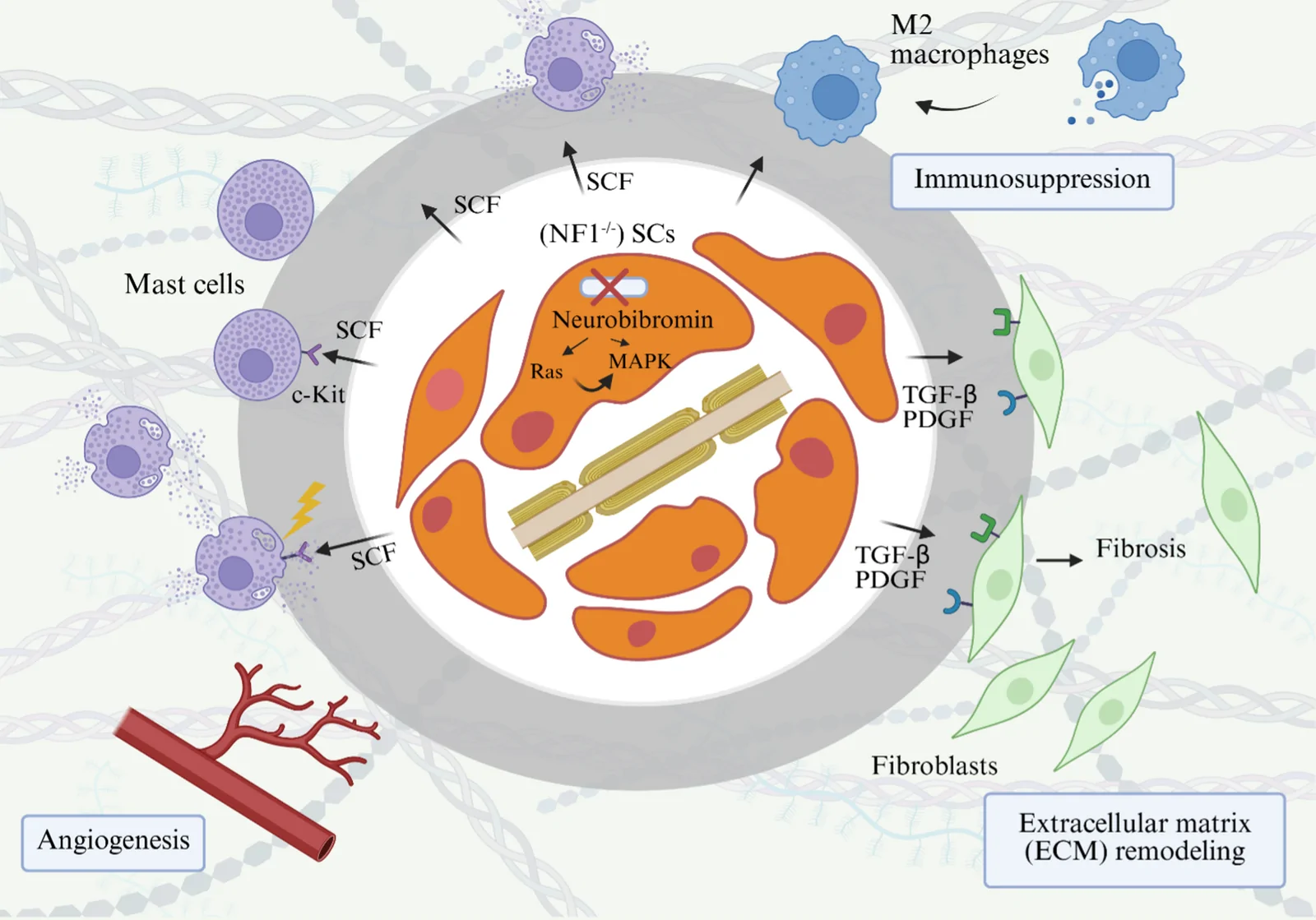
Overview of tumor–microenvironment interactions in NF1-associated neurofibroma. Loss of neurofibromin in NF1-deficient Schwann cells activates Ras/MAPK signaling and promotes secretion of stem cell factor (SCF), leading to mast cell recruitment and activation. Tumor-derived TGF-β and PDGF stimulate fibroblasts, driving fibrosis and extracellular matrix remodeling, while macrophages adopt an M2 phenotype that supports immunosuppression. Angiogenesis further sustains tumor growth, highlighting the microenvironment-dependent nature of NF1 tumor progression

Conditional, lineage-specific deletion studies further verify the concept that neurofibromatosis is governed by both cell-autonomous and non-cell-autonomous mechanisms, featuring distinct neural and non-neural compartments that contribute to tumor development and disease-associated tissue remodeling [71, 74, 79] (Fig. 2). Animal models have helped establish that NF1 pathogenesis involves coordinated contributions from both neuronal and non-neuronal cell populations [71, 72, 74, 79]. In NF1, neurofibromin dysregulation is amplified through interactions with the TME, encompassing stromal, immune, and ECM components that shape tumor behavior [3, 80]. Consequently, NF1-associated tumors are best understood as microenvironment-dependent disease systems rather than purely tumor cell-intrinsic neoplasms. This concept is consistently supported by animal model studies, particularly in plexiform neurofibromas and optic pathway gliomas [5, 71, 74, 79, 81–83]. Mammalian systems, particularly the neurofibroma and optic pathway glioma mouse models, have clearly demonstrated the dependence of tumor growth on specific microenvironmental components [52, 74, 82].

Neurofibromas develop within a conducive niche created by neoplastic Schwann cells, fibroblasts, macrophages, mast cells, vascular elements, and ECM [71, 82]. In optic pathway glioma models, microglia function as central mediators, with tumor growth requiring microglia-derived chemokines, such as CCL5 and CXCL12 [83–85]. These interactions extend beyond tumor expansion, as microglia also contribute directly to axonal injury and neurological dysfunction, demonstrating that clinical NF1 phenotypes are inseparable from microenvironmental signaling [83–85].

Evidence from alternative vertebrate systems illustrates this principle at the level of histological interactions. In zebrafish models carrying mutations in the *nf1* orthologs *nf1a* and *nf1b*, loss of neurofibromin affects not only tumor susceptibility but also tumor–host interaction patterns [65]. NF1-associated gliomas arising in *nf1a*^⁺/⁻^; *nf1b*^⁻/⁻^; *p53*-deficient zebrafish exhibit diffuse single-cell infiltration into adjacent brain parenchyma and lineage heterogeneity, underscoring the importance of tumor–tissue interactions and spatial context [65]. Although these models do not fully recapitulate the mammalian immune and stromal compartments, they feature conserved, microenvironment-relevant properties of NF1-deficient tumors, including invasive growth patterns and interactions with surrounding neural tissue [65].

The conditional deletion of *NF1* in Schwann cells is sufficient to initiate tumorigenic programs; however, sustained tumor growth and malignant progression depend on cooperative signaling from surrounding stromal and immune compartments [86, 87]. This system-level effect is also demonstrated in non-neoplastic contexts, such as skeletal models, in which cell type-specific *Nf1* loss disrupts local tissue homeostasis through indirect cellular interactions [55, 56]. Together, these findings highlight that loss of neurofibromin disrupts tissue homeostasis at the level of multicellular systems rather than acting solely through tumor cell-intrinsic mechanisms.

Stromal fibroblasts are a key functional component of the neurofibromatosis TME. In NF1 mouse models, fibroblasts and cancer-associated fibroblasts (CAFs) actively promote ECM deposition and remodeling, thereby reshaping tumor architecture and mechanical properties while promoting tumor expansion and invasion through paracrine signaling pathways [73, 78]. Experimental manipulation of ECM components in vivo has further demonstrated that structural proteins, such as collagen, function not only as passive scaffolds but also as active regulators of tumor–stromal interactions [88].

Immune cells constitute a functionally active component of the neurofibromatosis TME. Studies in NF1 mouse models demonstrate that macrophages and mast cells are actively recruited to tumor sites and participate in tumor-supportive signaling networks [75, 76, 82]. Macrophage polarization toward immunosuppressive phenotypes facilitates immune evasion and tumor persistence, whereas mast cell-derived mediators promote Schwann cell proliferation and survival [75, 76, 78, 82]. These immune–tumor interactions have been described primarily through in vivo animal models that enable the selective manipulation of immune cell populations and longitudinal assessment of tumor progression [74–76, 82]. At the molecular level, such interactions are partly mediated through defined paracrine signaling pathways, including KIT ligand-dependent mast cell activation and transforming growth factor β (TGF-β)-driven stromal and immunomodulatory signaling [78]. This dependency is most directly validated by bone marrow transplantation experiments, which demonstrate that stable neurofibroma development is unsustainable in the absence of specific hematopoietic cell populations, providing strong causal evidence for the requirement of the TME [74].

ECM remodeling represents another unifying theme across NF animal models [73, 88, 89]. Collagen-rich matrices are not merely structural features of neurofibromas but also actively regulate tumor growth, angiogenesis, and invasion [73, 88, 89]. At the molecular level, collagen VI, which is predominantly produced by fibroblasts, has emerged as a pro-tumorigenic ECM component that actively supports matrix remodeling and tumor maintenance in NF1-associated neurofibromas [89]. Hyaluronic acid (HA) further modulates tumor–stroma interactions by influencing cell proliferation, immune recruitment, and paracrine signaling [90]. Experimental systems enable the controlled interrogation of these matrix components, revealing that ECM remodeling is an early and integral event in tumor development, not a late consequence of expansion [3].

Soluble cytokines and growth factors represent another key regulatory layer within the neurofibromatosis TME. In animal models, TGF-β and other inflammatory signaling pathways regulate fibroblast activation, immune-cell behavior, and tissue remodeling [75, 77, 78]. Functional interrogation in mouse models has further demonstrated that dysregulated cytokine signaling contributes to tumor-associated inflammation [75, 77].

Recent conditional mouse models offer further temporal resolution, demonstrating that microenvironmental alterations precede overt tumor formation [49]. The Prss56-Cre knock-in line places Cre recombinase under endogenous Prss56 regulatory control and targets transient neural crest-derived boundary cap cells at the CNS–PNS interface and their descendants, which contribute to peripheral and cutaneous Schwann-cell lineages [49]. In Prss56-Cre;*Nf1*fl/fl conditional knockout mice, early microscopic cutaneous neurofibromas progressively incorporate non-mutant fibroblasts and mast cells and show increased vascularization and collagen-rich extracellular matrix remodeling [49]. Injury-induced models combined with transcriptomic analyses further establish inflammatory signaling as a central driver of the TME in NF, with strong activation of cytokine–chemokine pathways, ECM interaction networks, and immune cell recruitment programs that closely mirror molecular features observed in human neurofibromas [3]. In parallel, hyaluronan accumulation has been documented in NF1-associated neurofibromas and linked to intraoperative tumor edema, identifying an additional ECM-related feature of the NF1 microenvironment [90].

Although TME–driven mechanisms are well established in NF1-associated tumorigenesis, corresponding evidence in NF2 remains comparatively limited. Most NF2 animal models have focused predominantly on tumor cell-intrinsic consequences of merlin loss, with relatively limited direct interrogation of microenvironmental contributions. Nevertheless, anatomically targeted orthotopic models are beginning to provide important insights into the interactions between NF2-associated tumors and their local tissue context [69, 70, 91]. In particular, vestibular schwannoma CPA implantation models support tumor growth within a site-specific neural and stromal milieu and facilitate the longitudinal assessment of vascular responses, tumor–stroma interactions, and neurological dysfunction [69, 70, 91]. Although these models cannot establish the primary microenvironmental driver of NF2 tumorigenesis, they underscore the value of orthotopic approaches for functionally integrating tumor behavior with local tissue context and treatment response.

**Fig. 3:**
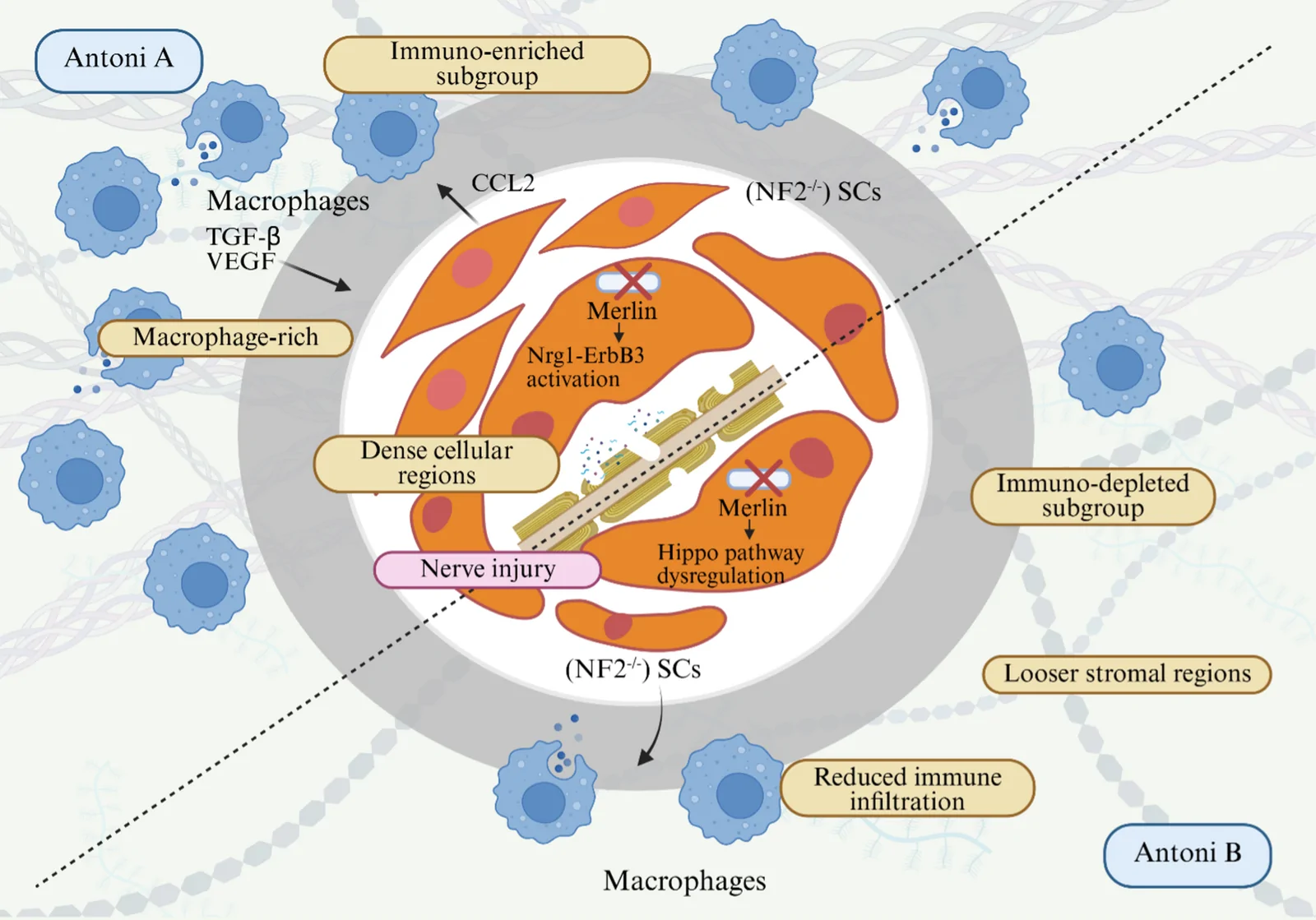
Microenvironmental heterogeneity in NF2-associated schwannoma. Merlin loss in NF2⁻/⁻ Schwann cells leads to Hippo pathway dysregulation and activation of Nrg1–ErbB3 signaling. In the setting of nerve injury, chemokine release (e.g., CCL2) promotes macrophage recruitment and sustained inflammation. The tumor displays spatial heterogeneity, with Antoni A regions characterized by dense cellularity and macrophage-rich, immune-enriched microenvironments, whereas Antoni B regions show looser stroma and reduced immune infiltration. These features highlight the interaction between merlin-deficient Schwann cells and a heterogeneous tumor microenvironment

Beyond these functional observations, direct experimental evidence has identified an active, causal role of the nerve microenvironment in NF2-associated schwannoma development [92] (Fig. 3). Using GEMMs combined with heterozygous *Nf2* loss in Schwann cells and axons, Schulz et al. demonstrated that alterations in the axonal microenvironment, particularly in nerve injury settings, significantly promote schwannoma formation [92]. This process was accompanied by sustained macrophage infiltration, unresolved inflammation, and marked defects in remyelination, indicating that persistent inflammatory and regenerative failure within the nerve microenvironment contributes to tumor development [92]. Together, these findings provide mechanistic support for a microenvironment-dependent component of NF2 schwannoma pathogenesis and complement orthotopic CPA models, which capture functional consequences of tumor–tissue interactions but do not directly interrogate microenvironmental drivers.

Recent studies have further refined this framework by demonstrating that microenvironmental heterogeneity in NF2-associated tumors is elicited by the intrinsic properties of merlin-deficient Schwann cells themselves [93]. Using a *Postn-Cre; Nf2*^*flox/flox*^ mouse model that develops synchronous NF2-mutant schwannomas, Chiasson-MacKenzie et al. found that intratumoral regions corresponding to Antoni A and Antoni B areas exhibit distinct patterns of immune cell infiltration, vascularization, and stromal organization [93]. These spatially defined paracrine microenvironments were linked to instability in intrinsic cell polarity following NF2 loss, leading to divergent programs of ErbB ligand production and polarized signaling [93].

The functional relevance of this tumor cell–microenvironment interplay is further supported by PDX studies of NF2-associated vestibular schwannomas [94]. In vivo PDX models derived from molecularly distinct NF2-SWN subgroups segregated into immune-enriched and immune-depleted microenvironmental states, which exhibited varying responses to targeted therapies [94]. Notably, blockade of colony-stimulating factor 1 receptor selectively suppressed tumor growth in immune-enriched models, providing direct evidence of the influence of microenvironmental composition on therapeutic vulnerability in a subset of NF2-associated schwannomas [94]. Collectively, these findings indicate that cell-intrinsic heterogeneity in NF2-mutant Schwann cells actively influences TME organization and composition, establishing a reciprocal and dynamic interaction between tumor cell state and local tissue context that provides meaningful implications for disease progression and treatment response (Fig. 3).

Collectively, these findings reveal the complexity of TME interactions in neurofibromatosis and underscore the indispensable role of experimental animal models in elucidating causal relationships. While species-specific differences must be considered, animal models remain essential for uncovering microenvironmental mechanisms that cannot be adequately addressed through human tissue studies alone.

## Conclusions

Neurofibromatosis is a complex, microenvironment-dependent disease in which genetic alterations induce tumorigenesis. However, sustained tumor growth, progression, and clinical manifestations are governed by dynamic interactions between neoplastic cells and their surrounding host tissues. Evidence derived from various animal models consistently demonstrates that neurofibromatosis-associated tumors cannot be adequately explained by tumor cell-intrinsic mechanisms alone, underscoring the critical role of the TME in shaping disease behavior.

Experimental animal models have helped establish causal relationships between tumor cells and microenvironment components, including immune populations, stromal cells, and ECM components. Through temporal- and cell type-specific genetic manipulation, immune modulation, and controlled stromal signaling disruption, these systems demonstrate the reliance of tumor maintenance and progression on sustained non-cell-autonomous interactions. Importantly, multiple models further demonstrate that microenvironmental alterations frequently precede overt tumor formation, further confirming the central role of the TME as an active driver rather than a secondary consequence of disease.

Despite their distinct experimental value, current animal models remain imperfect representations of human neurofibromatosis. In particular, species-specific differences in immune composition, stromal biology, and tumor kinetics necessitate careful model selection and cautious interpretation. Future refinement of experimental systems, such as approaches that better capture temporal dynamics, tissue injury responses, and microenvironmental heterogeneity, will be essential for advancing mechanistic insights. Recent reviews of targeted therapies for NF2-associated vestibular schwannomas highlight the expanding repertoire of molecular targets under investigation, while simultaneously emphasizing the absence of effective medical treatments. Furthermore, parallel assessments of preclinical modeling strategies underscore persistent limitations in experimentally capturing tumor-associated neurological deficits. Together, these observations reinforce the critical role of experimentally tractable and functionally integrated animal models in facilitating mechanistic discovery and clinical translation [95, 96]. Findings from animal models collectively confirm a conceptual shift toward viewing neurofibromatosis-associated tumors as ecosystem-level pathologies, providing an evidence-based foundation for therapeutic strategies that target not only tumor cells but also their supportive microenvironment, while emphasizing the continued importance of animal models in bridging fundamental biology with translational and clinical research.

## Data Availability

Not applicable. This article is a review and does not report original data.

## Abbreviations

3Rs: Replacement, Reduction, and Refinement
AKT/mTOR: Protein kinase B / mammalian target of rapamycin
CAFs: Cancer-associated fibroblasts
CCL5: C-C motif chemokine ligand 5
CDKN2A: Cyclin-dependent kinase inhibitor 2 A
CPA: Cerebellopontine angle
CXCL12: C-X-C motif chemokine ligand 12
CXCL8: C-X-C motif chemokine ligand 8 (Interleukin 8)
ECM: Extracellular matrix
EMT: Epithelial–mesenchymal transition
GAP: GTPase-activating protein
GEMMs: Genetically engineered mouse models
GGFβ3: Glial growth factor beta 3
HA: Hyaluronic acid
MPNSTs: Malignant peripheral nerve sheath tumors
NF1: Neurofibromatosis type 1
NF2: Neurofibromatosis type 2
NRG1: Neuregulin 1
P0/Mpz: Myelin protein zero
PAKs: p21-activated kinases
PDGF: Platelet-derived growth factor
PDX: Patient-derived xenografts
PI3K/AKT: Phosphatidylinositol 3-kinase / protein kinase B
Ras/MAPK: Ras/mitogen-activated protein kinase
SCF: Stem cell factor
TGF-β: Transforming growth factor beta
TME: Tumor microenvironment
TNF-α: Tumor necrosis factor alpha
VEGF: Vascular endothelial growth factor
